# Skeletal Effects of Early-Life Exposure to Soy Isoflavones—A Review of Evidence From Rodent Models

**DOI:** 10.3389/fped.2020.00563

**Published:** 2020-09-11

**Authors:** Kok-Yong Chin, Kok-Lun Pang

**Affiliations:** ^1^Department of Pharmacology, Faculty of Medicine, Universiti Kebangsaan Malaysia, Kuala Lumpur, Malaysia; ^2^State Key Laboratory of Oncogenes and Related Genes, Department of Urology, Renji-Med X Clinical Stem Cell Research Center, School of Medicine, Ren Ji Hospital, Shanghai Jiao Tong University, Shanghai, China

**Keywords:** bone, daidzein (CID 5281708), genistein (CID 5280961), neonates, life cycle, imprinting, perinatal, diethylstilbestrol (DES)

## Abstract

Isoflavones are dietary phytoestrogens commonly found in soy-based products. The widespread presence of isoflavones in soy infant formula and breast milk may have long-lasting effects on the development of sex hormone-sensitive organs like the skeleton. Animal early-life programming models are suitable for testing the skeletal effects of pre- and neonatal exposure of soy isoflavones. This review aims to collate the impacts of early-life exposure of soy isoflavones as evidenced in animal models. The isoflavones previously studied include daidzein, genistein, or a combination of both. They were administered to rodent pups during the first few days postnatal, but prolonged exposure had also been studied. The skeletal effects were observed when the animals reached sexual maturity or after castration to induce bone loss. In general, neonatal exposure to soy isoflavones exerted beneficial effects on the skeletal system of female rodents, but the effects on male rodents seem to depend on the time of exposure and require further examinations. It might also protect the animals against bone loss due to ovariectomy at adulthood but not upon orchidectomy. The potential benefits of isoflavones on the skeletal system should be interpreted together with its non-skeletal effects in the assessment of its safety and impacts.

## Introduction

Developmental programming or imprinting refers to the phenomenon whereby changes in the early development of an organism can exert long-lasting impacts that manifest during adulthood (1). Antenatal glucocorticoid use among pregnant women at risk of preterm delivery to assist the maturation of lungs of newborns is an example (2). Recent studies suggest the development of metabolic and cardiovascular abnormalities during adulthood in the offspring of mothers receiving glucocorticoids (3). This phenomenon has been replicated in primate models recently, whereby antenatal glucocorticoid exposure led to obesity in adult male baboons (4). The prevailing theory about the mechanism of developmental programming is epigenetic regulation by DNA methylation and histone modification induced by various factors (5).

The skeletal system is responsive to hormones and hormone-like substances. Prenatal and neonatal exposure of the skeletal system to hormonal stimuli might alter the trajectory of skeletal development. A single dose of estrogen at the first postnatal day could increase the bone mineral density (BMD) and cortical thickness of male mice at the prepubertal stage, but the inverse happened at the stage of peak bone mass acquisition (6). Prenatal and postnatal exposure of bisphenol A, a synthetic xenoestrogen, lowered the femoral bone stiffness of female rats but not in males (7). These studies highlight that early exposure of xenoestrogens can potentially impact bone development.

Isoflavones are phytoestrogens present abundantly in soy-based products (8) and are also an important source of dietary xenoestrogens to human infants. The average isoflavone content in soy infant formula ranges from 25 to 28 mg/100 g of powder, consisting of daidzein (DAI), genistein (GEN), and glycitein (9). Additionally, these isoflavones are also present in breast milk (10). Setchell et al. reported that circulating isoflavones of infants were 13,000–22,000 times higher than the estradiol level, and the exposure level was 6–11 times higher than the dose in adults with regular soy food intake (11). The American Academy of Pediatrics Committee on Nutrition only recommends soy formula to infants with inherited galactosemia, lactose intolerance, or from vegan families (12). Soy formulation is not recommended to infants with cow milk protein allergy because some of them also experience allergy to soy protein (12). A study in the United States showed that 11.6% of the infants ≤ 12 months consuming formula were being fed with soy-based products (13). The prevalent use of soy infant formula that may imply the reason to consume these products is based on preference rather than medical reasons. Other committees around the world take a stricter stand against the use of soy infant formula over concerns of the biological activities of phytoestrogens (14).

The skeletal effects of soy isoflavones take decades to develop, and the planning of epidemiological studies or interventional trials on this topic is difficult. Animal models of prenatal and neonatal programming enable us to have a glimpse of the potential effects of soy isoflavones on skeletal developments. Animals like rodents have a relatively short life cycle, so developmental changes can be examined within a shorter time. The purpose of this review is to examine the skeletal effects of early-life exposure to soy isoflavones in animal models of prenatal and neonatal programming.

## Animal Models

In toxicity testing, the neonatal life stage refers to the period from birth to 3 weeks of age (15), but the definition is debatable because newborn rodents are very immature due to the short gestation period, and some researchers suggest they better resemble human preterm infants (16). A similar definition has been used for rats (17). Thus, this review will focus on rodent models supplemented with soy isoflavones within this period. We noted that most neonatal and prenatal studies are the collective work of one research group, so the reader should take note of potential publication bias.

Most studies examining neonatal exposure of soy isoflavones on the skeletal system used mice as the model organism. The soy isoflavones studied included DAI, GEN, or a combination of both. Since the binding affinity of different types of isoflavones on biological targets, like estrogen receptors (18), are different, this could contribute to the variation of the results among the studies. Notably, a study reported that skeletal effects of a combination of GEN and DAI were less prominent compared to individual isoflavones (19), suggesting that the less active isoflavone could attenuate the effects of the more potent isoflavone.

The soy isoflavones were administered subcutaneously during the first few postnatal days, and the skeletal outcomes were assessed when the mice reached maturity (4 months of age), with diethylstilbestrol (DES) as the positive control. The dose and type of isoflavone used in each study were different [GEN, 4 μg/day sc or 5 mg/kg body weight (bw)/day sc; DAI, 2 mg/kg bw/day sc; GEN + DAI, 7 mg/kg bw/day sc]; thus, the skeletal effects may not be comparable between studies. Other variations of the models exist. Two studies assessed the skeletal effects of prenatal exposure of folic acid (FA) and postnatal exposure of isoflavones on mice (20, 21); thus, the effects of isoflavones were confounded by prenatal FA treatment.

In the prenatal and postnatal rat model (22, 23), soy isoflavones were administered through food, in contrast to subcutaneous for studies in mice. Soy isoflavones like DAI are known to be metabolized by gut microbiomes to equol and O-desmethylangolensin with different biological activities compared to the parent compounds (24). However, in all studies reviewed, the bioavailability of the isoflavones and their metabolites were not measured. Along with the doses that were not readily translatable between the mouse and rat studies, these factors prevent effective comparisons between different research groups.

Another study exposed the mouse pups with isoflavones, castrated them in the fourth month, and assessed their bone health status in the eighth month (25). The castration model is a classic bone loss model due to sex hormone deficiency (26), so the mentioned study examined the antiosteoporosis effects of early exposure to isoflavones.

The composition of the control diet is essential to ensure a fair comparison between the isoflavone-supplemented and control groups. Long-term supplementation of different diet regimens with and without soy isoflavones or protein can modify body composition and metabolic profile of the animals (27). Most studies indicated the use of soy-free diet. Modified AIN93G diet without isoflavones is the most common diet used (19, 23, 28), while other studies indicated the use of diet without DAI (23) or isoflavones (22), or amino acid-based diet without isoflavones (20, 21). The difference in the composition of these diets could cause variation in the results of the studies reviewed.

The design of the studies is shown in Table 1 for comparison.

**Table 1:** Skeletal effects of prenatal and/or neonatal exposure of soy isoflavones derived from animal studies.

**Study**	**Study design**	**Bone mineral density**	**Bone microstructure**	**Bone strength**	**Bone remodeling and biochemical markers**
Peikarz and Ward (28)	CD-1 mouse pups were exposed to corn oil, GEN (4 μg/day sc), or DES (2 μg/day sc) for the first 5 days of life. Outcomes measured at 4 months of age. Control diet: AIN93G diet without ISO	Male Femoral BMD: GEN = corn oil < DES Lumbar BMD: GEN > DES = corn oil	NA	Male Femoral peak load: GEN = corn oil < DES Lumbar peak load: GEN > DES = corn oil	Male Serum osteocalcin: GEN = DES < corn oil Serum CTX-1: NS
		Female Femoral BMD: GEN = DES > corn oil Lumbar BMD: GEN = DES > corn oil	NA	Female Femoral Peak load: NS Lumbar peak load: GEN = DES > corn oil	Female Serum osteocalcin & CTX-1 NS
Kaludjerovic and Ward (19)	CD-1 mouse pups were exposed to corn oil, DAI (2 mg/kg bw/day sc), GEN (5 mg/kg bw/day sc), DAI + GEN (ISO, 7 mg/kg bw/day sc), or DES (2 mg/kg bw/day sc) for the first 5 days of life. Outcomes measured at 4 months of age. Control diet: AIN93G diet without estrogenic compounds.	Male Lumbar BMD: DES = all isoflavones group > corn oil Femoral midpoint BMD: DAI > GEN = ISO = DES = corn oil	NA	Male Lumbar peak load: NS Femoral midpoint yield: NS Femoral midpoint peak load: DES < DAI = ISO, DES = corn oil = GEN Femoral midpoint stiffness: NS Femoral neck yield: DAI > DES, ISO > DES, corn oil = DES = GEN Femoral neck peak load: DAI > corn oil = GEN = ISO ≥ DES Femoral neck stiffness: NS	NA
		Female Lumbar BMD: DES > all isoflavones group = corn oil Femoral midpoint BMD: DAI > GEN = ISO = DES = CON	Female ***Lumbar*** BV/TV: DAI = DES > corn oil > GEN, ISO = corn oil = GEN BS/BV: DAI > all groups Tb/Th: GEN = ISO > corn oil = DAI = DES Tb.N: DAI = GEN = DES > corn oil = ISO Tb.Sp: DAI = DES > GEN = ISO > corn oil ***Femoral neck*** BV/TV: DAI = GEN > corn oil = ISO = DES BS/BV: DES > DAI, = other groups, DAI = other groups Tb/TH: DAI > DES = corn oil = GEN = ISO Outer cortical perimeter: DAI > corn oil = DES = GEN = ISO Cortical area: DAI > corn oil = ISO, DAI > DES, DES = corn oil Marrow area: GEN = corn oil > DES, GEN = DAI, DES = DAI, DAI = ISO ***Femoral midpoint*** Tb.Sp: DAI = GEN > DES, DES = ISO = corn oil Cortical area: DAI > corn oil = DES, ISO = GEN = DES = corn oil, ISO = DAI Cortical thickness: DAI > corn oil = DES, DAI = GEN = ISO	Female Lumbar peak load: DAI = DES > ISO = corn oil Femoral midpoint yield: DES > all isoflavones = corn oil Femoral midpoint peak load: corn oil < DES > GEN = ISO, isoflavones > corn oil Femoral midpoint stiffness: corn oil < all isoflavones, DES = DAI > GEN = ISO Femoral neck yield, peak load, stiffness: NS	NA
Kaludjerovic and Ward (25)	CD-1 mouse pulps were exposed to corn oil, DAI + GEN (ISO, 7 mg/bw/day sc), or DES (2 mg/bw/day sc) for the first 5 days of life. Castration was performed at 4th month. Outcomes measured at 8th month of life. Control diet: AIN93G diet	Male Lumbar BMD: NS Whole femur BMD: NS	NA	Male Lumbar peak load: NS Femoral midpoint peak load, yield, stiffness: NS Femoral neck peak load: ISO > DES, DES = corn oil Femoral neck yield, stiffness: NS	NA
		Female Lumbar BMD, BMC: ISO > corn oil = DES Whole femur BMD: ISO > corn oil, = DES, corn oil = DES	Female ***Lumbar*** BV/TV: NS Tb/Th: DES = ISO > corn oil Tb.N: DES > corn oil, ISO = DES = corn oil Tb.Sp: NS ***Femoral neck***: All NS	Female Lumbar peak load: DAI + GEN > corn oil = DES Femoral midpoint peak load: ISO > corn oil = DES Femoral midpoint yield, stiffness: NS Femoral neck peak load, yield, stiffness: NS	NA
Hertrampf et al. (22)	Sprague–Dawley female rats Dams fed with diet without isoflavones (IDD), mixed isoflavone diet (IRD: GEN 240 μg/g + DAI 232 μg/g), or GEN (700 μg/g diet). Pups fed with the same diet as dams for 80 days postnatal^*^	Tibial BMD at Day 21: GEN = IRD > IDD	NA	NA	NA
Kaludjerovic and Ward (20)	CD-1 mouse dams fed with low (0 mg/kg bw/day), adequate (2 mg/kg bw/day) and supplementary (8 mg/kg bw/day) FA. Pups fed with either corn oil or DAI + GEN (ISO, 7 mg/kg bw/day, sc) from day 1–10 after birth. Outcomes measured at 4 months of age. Only male pulps are studied. Control diet: amino acid-based diet without ISO	Male Lumbar BMD: Overall ISO > no ISO Whole femur BMD: ISO + adequate FA group > FA only	NA	Male Lumbar peak load: Overall ISO > no ISO Whole and midpoint femur peak load: ISO + adequate FA group > FA only	Male Serum biomarkers OPG and OPG/RANKL ratio: ISO + supplementary FA group > FA only
Kaludjerovic and Ward (21)	CD-1 mouse dams fed with low (0 mg/kg bw/day), adequate (2 mg/kg bw/day), and supplementary (8 mg/kg bw/day) FA. Pups fed with either corn oil or DAI + GEN (ISO, 7 mg/kg bw/day, sc) from days 1–10 after birth. Outcomes measured at 4 months of age. Only female pulps are studied. Control diet: amino acid based diet free from ISO	Female Femur and lumbar BMD: adequate FA + ISO group > FA only	Female Lumbar BV/TV, Tb.N: Overall ISO > no ISO Lumbar Tb.Sp: Overall ISO < no ISO Femur Tb.Th, Tb.N: Adequate FA + ISO > FA only	Female Lumbar peak load: NS Femur midpoint peak load: ISO + adequate FA group > FA only	Female OPG, OPG/RANKL, IGF-1: Adequate FA + ISO > FA only Dnmt3a mRNA: Adequate FA + ISO < FA only; Supplemental FA + ISO > FA only NPY mRNA: Adequate FA + ISO < FA only
Tousen et al. (23)	Control: Sprague–Dawley dams and offspring fed on DAI-free diet. D1: Sprague–Dawley dams and offspring fed with DAI (0.5 g/kg diet) till postnatal day 13^**^	Male ***Whole femur BMD*** Day 22: D1 = control Day 35: D1 < control Day 77: D1 < control	NA	NA	Male ***Serum osteocalcin*** Day 22: D1 < control Day 35: D1 = control Day 77: D1 = control
		Female ***Whole femur BMD*** Day 22: D1 = control Day 35: D1 < control Day 77: D1 = control	NA	NA	Female ***Serum osteocalcin*** Day 22: D1 = control Day 35: D1 < control Day 77: D1 = control

*BMD, bone mineral density; BV/TV, bone volume; bw, body weight; CTX-1, C-terminal telopeptide of type 1 collagen; DAI, daidzein; DES, diethylstilbestrol; Dmnt3a, DNA methyltransferase 3a; FA, folic acid; GEN, genistein; IDD, dams fed with diet without isoflavones; IGF-1, insulin-like growth factor-1; IRD, dams fed with mixed isoflavones diet (GEN + DAI); ISO, isoflavone mixture (GEN + DAI); NA, not available; NS, not significant among all the groups; NPY, neuropeptide Y; OPG, osteoprotegerin; RANKL, receptor activator of nuclear factor kappa-B ligand; Tb.N, trabecular number; Tb.Th, trabecular thickness; Tb.Sp, trabecular separation; sc, subcutaneous*.

* *Only data till day 21 is included in this table*.

** *There was another group fed till postnatal day 77*.

## Neonatal Exposure

### Effects on Bone Mass

Bone mineral density (BMD) assessment through dual-energy X-ray absorptiometry in animals allows non-invasive longitudinal changes in bone mass (29). Neonatal exposure to GEN increased the femoral BMD of mice at sexual maturity. Additionally, it also increased the lumbar BMD of female mice (28). The effects of GEN was greater than DES in males but on par with DES in female mice (28). In another study, DAI was more potent than GEN, DAI + GEN, and DES in increasing the femoral midpoint BMD of mice (19). However, DES exerted greater effects on female, while all treatments exerted similar effects on male lumbar BMD (19). In prenatal FA and postnatal isoflavone (DAI + GEN) models, femoral and lumbar BMD increased significantly in male mice exposed to adequate FA (2 mg/kg bw/days) and postnatal isoflavone compared to other treatment groups (20). Similar treatment resulted in higher lumbar and femoral BMD of the female mice (21). Neonatal isoflavone exposure (DAI + GEN) also prevented deterioration of lumbar and femoral BMD due to castration in female rats but not in male rats (25).

Overall, neonatal exposure to isoflavones increased BMD of the mice at sexual maturation, and the effects were slightly better in male mice. However, it did not protect male mice against bone loss due to sex hormone deficiency. These observations suggest that both androgenic and estrogenic stimulation are essential in the development of the skeleton in males (30, 31). On the other hand, the adverse effects of androgen deprivation on bone are tremendous and cannot be displaced by earlier BMD gain caused by soy isoflavones.

BMD cannot differentiate between the trabecular and cortical compartment, and it has a low resolution (8). Microcomputed tomography assessment, which is discussed below, provides a more complete picture of the microstructure in each bone compartment.

### Effects on Bone Microarchitecture

Skeletal microarchitecture is a major determinant of bone strength, which can be studied through microcomputed tomography (32). Neonatal exposure of DAI significantly improved the trabecular microstructure indices of the lumbar spine and femoral neck, and the trabecular separation of the femoral midpoint in female mice (19). The effects of GEN were superior to those of DAI on lumbar trabecular thickness (19). Similarly, neonatal isoflavone exposure independently increased lumbar trabecular microstructure in female offspring from dams supplemented with FA during gestation (21).

On cortical bone indices, DAI increased the cortical area of the femoral neck and midpoint (19). DAI, GEN, and DAI + GEN also improved cortical thickness of the midpoint but not at the femoral neck (19).

Overall, data on neonatal exposure of isoflavones on bone microarchitecture is limited. The available study showed that improvements were site specific. The lumbar spine and femoral neck are trabecular rich; therefore, the improvement in trabecular indices is better. The femoral midshaft is cortical rich; hence, cortical improvement is more apparent. The variations in bone composition could significantly influence the effects of isoflavones on different bone regions, which dictates bone strength.

### Effects on Bone Strength

The ultimate measure of bone health is skeletal strength, which predicts fracture risk. The usual indices include ultimate/peak load, which refers to bone strength under certain specific loading conditions, and stiffness, which refers to the resistance of the bone against deformation (33). Neonatal GEN significantly increased the lumbar, but not the femoral, peak load of adult male mice compared to DES, and the normal control (28). The effects of neonatal GEN and DES exposure on lumbar peak load were similar in adult female mice, whereby an improvement over normal control was observed (28). Both compounds did not alter the femoral peak load in adult female mice (28). Since the lumbar spine is rich in trabecular bone, the improvement in strength could be a result of enhanced trabecular bone structure.

On the other hand, neonatal DAI exposure increased the femoral neck and midpoint peak load in adult male mice but not in female mice (19). The effects of DAI were greater than those of DES in male mice, while the effects of GEN or DAI + GEN were intermediary (19). Neonatal isoflavone exposure only retained the peak load of the femoral neck in male mice and the peak load of the femoral midpoint in female mice after castration (25). Adequate prenatal FA and postnatal isoflavone exposure increased the lumbar and femoral peak loads in male mice (20). Contradictory to BMD results, the femoral midpoint, but not the lumbar peak load, was higher in female mice treated with adequate prenatal FA + postnatal isoflavone (21). The beneficial effects on cortical-rich regions in these studies due to DAI or isoflavone mixture with DAI correspond to the results of skeletal microstructure, whereby DAI increased cortical thickness. It also warrants further investigation on whether the skeletal action of DAI is selective on cortical bones.

Overall, neonatal isoflavone exposure generally improves the bone strength of mice, but the skeletal sites vary due to the type of isoflavones present.

### Effects on Bone Remodeling

Bone remodeling refers to the tightly regulated process of bone resorption by osteoclasts and formation by osteoblasts (34). The bone remodeling process can be inferred by circulating markers of bone resorption and formation, which are the products of bone cell activities (35). Neonatal GEN exposure did not change the serum C-terminal telopeptide of type I collagen (CTX-1; bone resorption markers) and osteocalcin level (bone formation markers) in mice (28). On the other hand, adequate prenatal FA plus postnatal isoflavone increased osteoprotegerin (OPG) and OPG/receptor activator of nuclear factor kappa-β ligand (RANKL) ratio in male mice (20) and female mice (21). Since OPG prevents the action of RANKL in stimulating osteoclast differentiation, this observation indicates the suppression of bone resorption (36). Additionally, the serum level of insulin-like factor-1, a bone anabolic hormone, was increased in female mice (21).

Overall, neonatal isoflavone exposure might suppress the formation of osteoclasts, but this observation was not reflected in the bone resorption markers. Perhaps this change is transient and has normalized upon adulthood.

## Models and Prenatal and Neonatal Exposure

Other studies reported a longer period of isoflavone exposure involving the prenatal and neonatal periods. We limit our discussion to studies with postnatal exposure within the limit of the neonatal period of life. Tousen et al. (23) reported on the skeletal effects of rat dams fed with DAI from gestation till postnatal day 13 and their offspring fed with control diet until weaning. The whole BMD of the male and female offspring was significantly lower at the prepubertal stage (day 35) compared to the control rats (23). Upon sexual maturation (day 77), the BMD of the male remained lower compared to that of the normal control, but the BMD of the female returned to control values (23). The serum osteocalcin level of the exposed rats was lower compared to that of the control rats at the prepubertal stage, but the level returned to control values upon sexual maturity (23). Another study reported a lower tibial BMD in female offspring of rat dams exposed to GEN or isoflavones during gestation and lactation period (postnatal 21 days) (22). These effects persisted when the rats reached adulthood, but it should be noted that the offspring were fed with the same diet as the dams' lifelong (22).

Overall, in contrast to neonatal exposure, prenatal plus postnatal exposure of isoflavones exerted negative effects to the bone. This observation might be related to the critical period of intrauterine exposure. It was reported that soy isoflavones could regulate the epigenetic process of embryonic stem cells (37). Whether these changes are beneficial or harmful to the development of the skeletal system remains a question.

**Figure 1:**
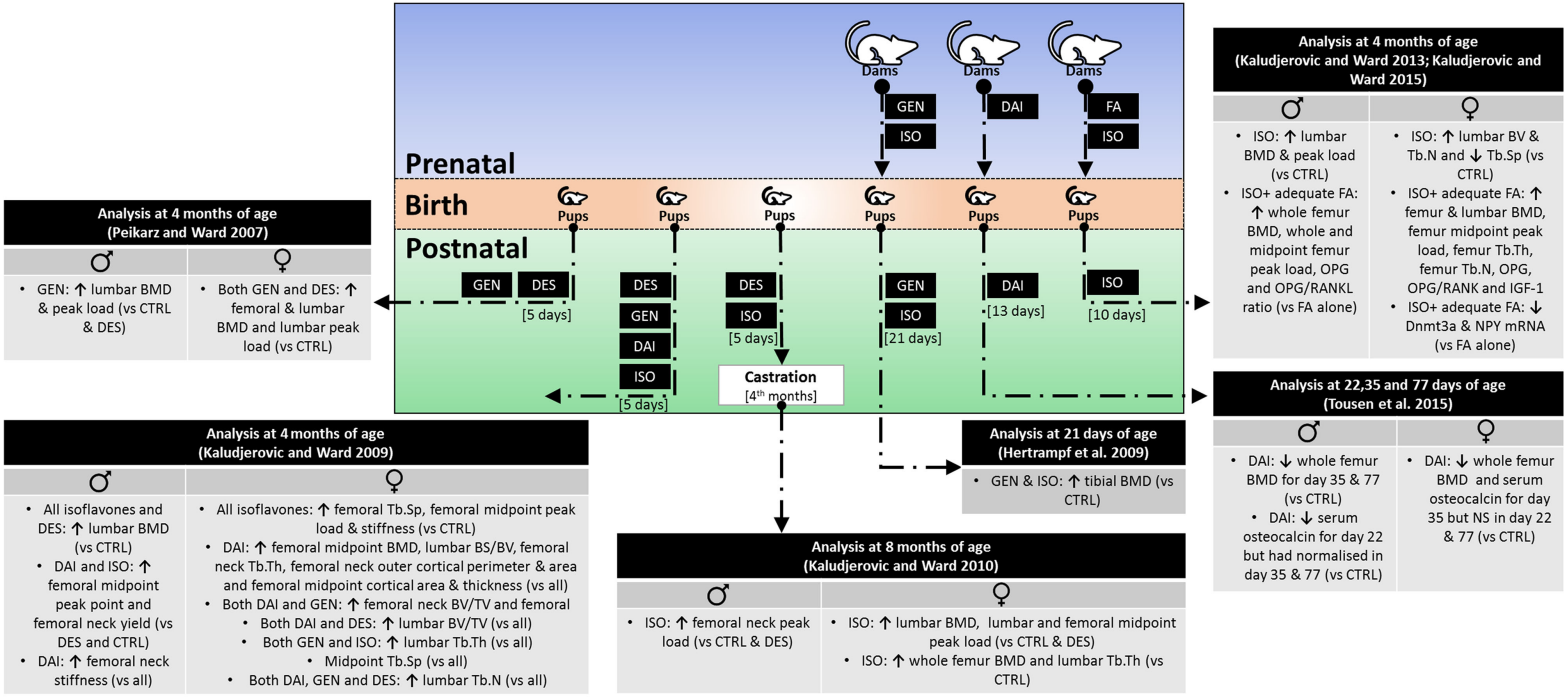
Skeletal effects of early life exposure to soy isoflavones. BMD, bone mineral density; BV/TV, bone volume over total volume; CTRL, control; DAI, daidzein; DES, diethylstilbestrol; Dnmt3a, DNA methyltransferase 3a; FA, folic acid; GEN, genistein; IGF-1, insulin-like growth factor-1; ISO, isoflavone mixture (DAI + GEN); NPY, neuropeptide Y; OPG, osteoprotegerin; RANKL, receptor activator of nuclear factor kappa-B ligand; Tb.N, trabecular number; Tb.Th, trabecular thickness; Tb.Sp, trabecular separation.

Current findings from animal experiments are summarized in Figure 1.

Prepubertal exposure of soy isoflavones also influences skeletal health at adulthood. Zhang et al. (38) showed that female rats fed with soy protein isolates for 14 days from postnatal day 20, had higher bone formation markers (alkaline phosphatase, ALP, and osteocalcin), bone resorption markers (RatLap), and sclerostin level (a negative regulator of bone formation) compared to the control. Chen et al. (39) compared the rats fed with casein or soy protein isolate-based diet starting from the postnatal day 24 till postnatal day 55 and subsequently switched to standard diet or continue with soy protein isolates till 6 months old. Some of the rats were castrated at the age of 6 months and sacrificed 1–3 weeks later. They reported that the total BMC and BMD, and trabecular and cortical BMD of the soy protein isolate group were greater than those of the casein group. After castration, rats on short-term or long-term soy protein isolates had better bone health than those on the casein diet. Overall, prepubertal exposure of soy isoflavones exerts skeletal beneficial effects similar to those of neonatal exposure models. In contrast, prenatal and postnatal exposures of soy isoflavones exert negligible or negative effects on rats in adulthood. However, apart from the period of exposure, the dose and type of isoflavones supplemented should be considered in interpreting the results of these studies.

Hotchkiss et al. (40) compared the skeletal effects of GEN in rats supplemented continuously for 2 years or switched to the control diet after postnatal day 140. Lifelong exposure to high-dose GEN resulted in lower lumbar BMC and area, as well as higher pyridinoline (a bone resorption marker) compared to those of rats supplemented with lower doses. In female rats treated till postnatal day 140, pyridonoline was lower and ALP was higher with higher GEN dose, indicating that GEN induced higher bone formation rate in these rats. In rats treated with GEN till weaning and male rats at all life stages, no skeletal effects were observed. The data may indicate that androgenic regulation is essential for skeletal development in males and that phytoestrogenic effects of isoflavones may not be as important as in females.

## Mechanism of Action of Isoflavones on Bone Development

Soy isoflavones affect skeletal development through direct and indirect action on bone cells. In this section, the actions of soy isoflavones on bone cells, through DNA methylation and thyroid hormones (TH), are discussed.

GEN exerts a positive effect on bone remodeling by reducing osteoclast and increasing osteoblast number. It lowers the osteoclast number in neonatal bone marrow cell culture through decreasing the survival of osteoclasts or attenuating their formation (41). It also increases the differentiation and migration of primary rat calvarial osteoblasts by estrogen receptor (ER) and nitric oxide synthase pathway (42). Mitogen-activated protein kinase (MAPK) and phosphoinositide 3-kinase (PI3K) transduction systems are shown to involve in the pro-osteogenesis effects of GEN (42). Surprisingly, in a coculture of osteoblasts and monocytes, GEN increased the formation of osteoclast-like cells (42), probably through stimulating factors from osteoblasts. Another study suggested the upregulation of *Ereg* and *Efcab2*, as well as downregulation of *Hrc, Gli*, and *Ifim5* in MC3T3-E1 preosteoblastic cells (43).

Similarly, DAI promoted apoptosis of osteoclast-like cells derived from porcine bone marrow cells by caspase-3 cleavage and upregulation of nuclear ER (44). DAI improved osteoblastic differentiation of OCT-1 cells via upregulation of bone morphogenetic protein-2 (BMP-2) and activation of the SMAD pathway (44). DAI also improved proliferation, differentiation of MG-63 osteoblasts primarily through ERα signaling and at least partly through MAPK kinase/extracellular regulated kinase (MEK/ERK) and the PI3K/protein kinase B (Akt) signaling pathway (45). Another study showed an increased expression of ERα, ERβ, and steroid receptor (SR) comprising the classic estrogen-responsive elements in MG-63 osteoblasts induced by DAI (46). It also reduced the expression of stimuli for osteoclastogenesis [RANKL and interleukin-6 (IL-6)] while increasing the expression of OPG in MG-63 osteoblasts (46).

Equol and O-desmethylangolensin, derived from DAI metabolism, could also inhibit the formation of osteoclast-like cells from bone marrow cells from male mice (47). Equol also promoted osteoblast differentiation from primary osteoblast precursors, probably through ER and the protein kinase C-α (PKC-α) pathway (48).

DNA methylation is an epigenetic process mediating gene expression, which plays an important role in skeletal development. It is regulated by DNA methyltransferase (Dnmt) (49). The differentiation of mesenchymal stem cells to osteoblastic or non-osteoblastic progenitors is linked to the methylation rate of critical genes encoding BMP-2 and ALP, which is responsive to the Wnt signaling pathway (50). Methylation of the gene encoding interferon regulatory factor eight facilitates osteoclastogenesis (51). Previous studies have demonstrated that the antiestrogenic effects of isoflavones could be partially attributed to its effects on the methylation of steroidogenic factor 1 (49, 52). Exposure of mouse embryonic tissues to GEN also modified the regulation level of genes (37). Since DNA methylation is the prevailing theory for neonatal imprinting effects, the effects of prenatal FA plus postnatal isoflavones on Dnmt in the bone for female mice have been explored. Postnatal isoflavone exposure decreased Dnmt3a expression in the adequate FA group but increased Dnmt3a expression in the supplemental FA group, indicating a significant interaction between FA and isoflavones (21). Gene expression analysis demonstrated that neuropeptide Y (NPY) level, a potent osteogenesis inhibitor, was suppressed in the adequate FA plus isoflavone group (21). Other epigenetic factors, like histone modifications, could also influence bone health, but the role of soy isoflavones in mediating bone health through this mechanism is less clear.

On the other hand, soy isoflavones exert their effects on the skeletal system indirectly through interference with TH homeostasis (53). Congenital and juvenile hypothyroidism can delay skeletal development, while thyrotoxicosis can accelerate skeletal aging and premature closure of growth plate. These phenomena have been replicated in genetically modified rodent models with altered thyroid hormone signaling (54). On the other hand, the skeletal effects of over hypo/hyperthyroidism and thyroid-stimulating hormone (TSH) are well-known (55, 56). Epidemiological studies showed that soy isoflavone consumption could influence TH levels. Intakes of soy isoflavones or protein are associated with high TSH, especially among women (57), which could be a result of the thyroid suppressive effect of isoflavones. In contrast, urinary isoflavone levels are found to be associated with higher serum thyroxine (T4) in women but not in men (58), in which the authors attribute the higher T4 to the suppressive effects of isoflavones on deiodination of T4 to T3. Mechanistically, soy isoflavones suppress the level of TH by inhibiting thyroid peroxidase (TPO) activity competitively (59) and inhibit the binding of TH to thyroid transport protein [transthyretin (TTR)] in the blood (60, 61). Soy isoflavones, especially GEN, are also deiodinase (Dio) inhibitors, specifically type 1 (Dio1) (62). However, Dio1 is not expressed in the bone (63). *In silico* analysis showed that isoflavones could bind to thyroid receptors, but this effect has not been validated *in vitro* (64). Isoflavones were reported to enhance transcription mediated by triiodothyronine-liganded thyroid receptor (64). Soy isoflavones also inhibit aromatase and 17-β-hydroxysteroid dehydrogenase (17-β-HSD) (65), which prevent the conversion of androgens to estrogens, thereby lowering thyroxine-binding globulin (TBG) and decreasing T4 level (66). However, TTR, instead of TBG, is the main carrier of thyroid hormones in rodent blood (67).

Soy isoflavones may also affect skeletal health through other indirect mechanisms. A multigeneration supplementation study showed that soy protein concentrates and soy isoflavones slowed down the weight gain and reduced the insulin level of the rats (27). The reduction in mechanical loading exerted by body weight and the bone anabolic insulin signaling may be harmful to the bone. Soy protein isolates also decreased the expression of caveolin-1, a membrane protein that anchors other proteins to the membrane, accompanied by activation of BMP-2/SMAD signaling in bone and osteoblasts (38). In another study, long-term soy protein isolate supplementation caused concurrent reductions in caveolin-1, p 53, p 21, and senescence-associated beta-galactosidase in the bone of rats and osteoblasts cultured with the serum of treated rats (39), thus, preventing senescence of osteoblasts. These are translated to a beneficial effect on the bone.

**Figure 2:**
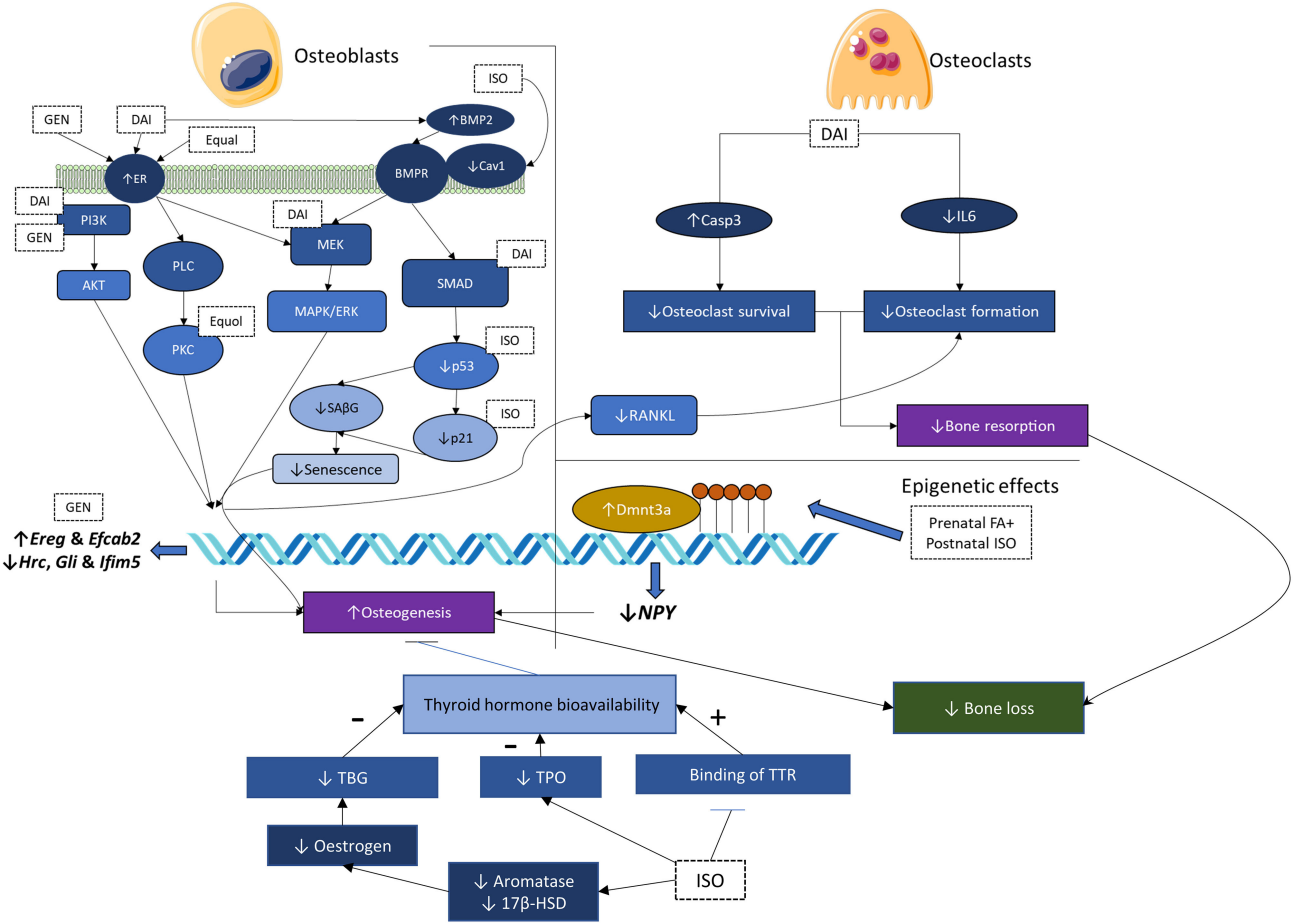
Mechanisms of action of soy isoflavones and soy protein isolates. 17-β-HSD, 17-β-hydroxysteroid dehydrogenase; BMP-2, bone morphogenetic protein-2; Cav1, caveolin-1; DAI, daidzein; Dnmt3a, DNA methyltransferase 3a; ER, estrogen receptor; ER-α/β, estrogen receptor-α/β; FA, folic acid; GEN, genistein; IL-6, interleukine-6; ISO, isoflavone mixture; MAPK, mitogen-activated protein kinase; MEK/ERK, MAPK kinase/extracellular-regulated kinase; NPY, neuropeptide Y; PI3K/Akt, phosphoinositide 3-kinase/protein kinase B; PKC, protein kinase C; PLC, phospholipase C; RANKL, receptor activator of nuclear factor kappa-B ligand; SAβG, senescence-associated beta-galactosidase; T4, thyroxine; TBG, thyroxine-binding globulin; TH, thyroid hormone; TPO, thyroid peroxidase; TTR, transthyretin.

Overall, soy isoflavones modulate a complex network of factors influencing bone health. The net effects seem to be beneficial, considering the phenotypic response of the skeletal system on soy isoflavone supplementation. The mechanisms of action of soy isoflavones and soy protein isolates are summarized in Figure 2.

## Conclusion

Early-life exposure to isoflavones, especially during the neonatal period, enhances BMD, skeletal microstructure, and strength in adulthood. Similarly, prepubertal or life-long supplementation suggested beneficial skeletal effects, which may reduce the skeletal negative effects of castration at adulthood. On the other hand, the prenatal plus postnatal exposure models reveal the negative effects of isoflavones. Further, basic research on the influence of isoflavones on the epigenetic process in embryos would explain the skeletal phenotypes observed. Mechanistically, the skeletal protection may be due to their direct actions on bone cells favoring osteogenesis and DNA methylation. The effects of isoflavones on TH homeostasis could bring more harm than benefits to the skeleton at this life stage. Therefore, soy infant formulation should not be initiated without proper evaluation of the risk and benefits. The evidence presented in this review should also be interpreted together with evidence from other organ systems, especially sex hormone-sensitive organs to assess the impacts of early-life exposure of isoflavones on developmental health holistically.

## Author Contributions

K-YC and K-LP conceived the review. K-YC performed the literature search and drafted the manuscript. K-LP validated the search results and reviewed the manuscript. All authors contributed to the article and approved the submitted version.

## Conflict of Interest

The authors declare that the research was conducted in the absence of any commercial or financial relationships that could be construed as a potential conflict of interest.
